# Supplementary material for: Accurate immune repertoire sequencing reveals malaria infection driven antibody lineage diversification in young children
Source: Nat Commun. 2017 Sep 14;8:531. doi: 10.1038/s41467-017-00645-x (PMC5599618; doi:10.1038/s41467-017-00645-x)
Supplement: Supplementary file 1 — Supplementary Information [file 41467_2017_645_MOESM1_ESM.pdf]

Supplementary Figures

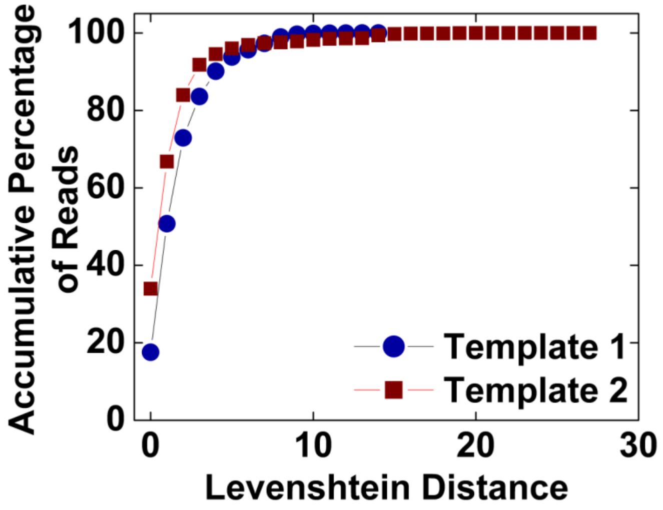

Supplementary Figure 1 Cumulative distribution of reads as a function of Levenshtein distance between RNA control templates and sequencing reads. The lengths of control templates and reads were 150bp. More than 99% of reads are similar to control templates under the Levenshtein distance of 23. Therefore we set the sub-group clustering threshold as 15% of the read length.

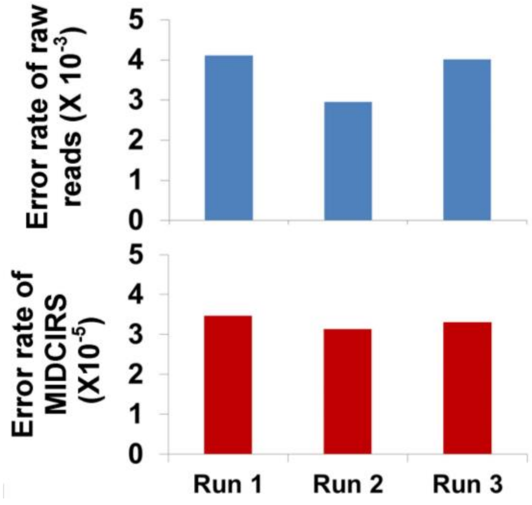

Supplementary Figure 2 Comparison between raw error rate and improved error rate after using MIDCIRS. Raw reads error rates (top, blue) and MIDCIRS consensus error rates (bottom, red) for 3 Miseq runs, calculated as described in **Methods**.

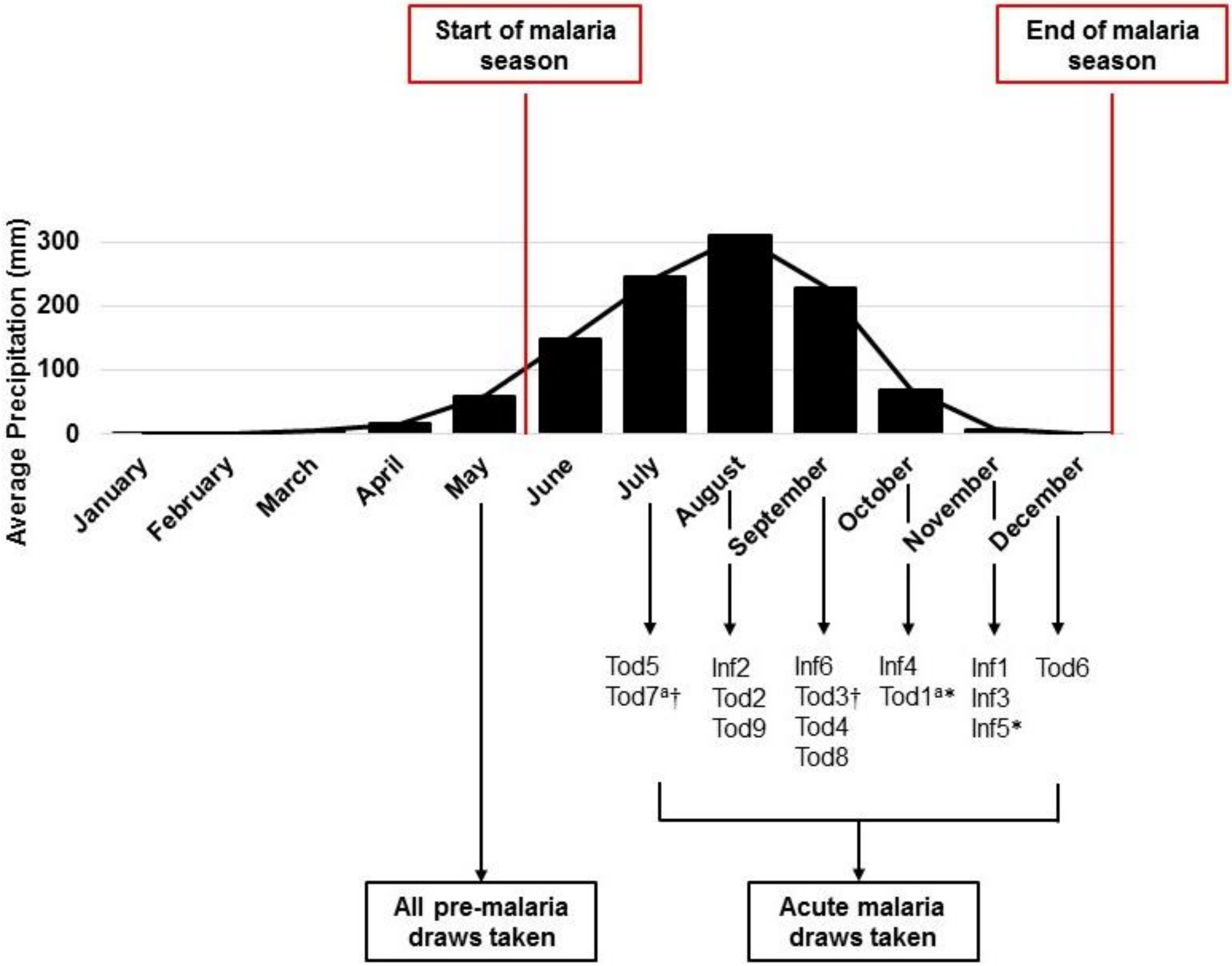

Supplementary Figure 3 Sample collection timeline. All pre-malaria blood draws were taken in May, just before the start of the rainy season. Acute malaria blood draws were taken 7 days after the onset of acute febrile malaria. Unless otherwise indicated ([a]), all samples were collected during 2011. Average precipitation was estimated from the neighboring city of Bamako, Mali (climatemps.com).

* Same individual
† Same individual
[a] Drawn in 2012

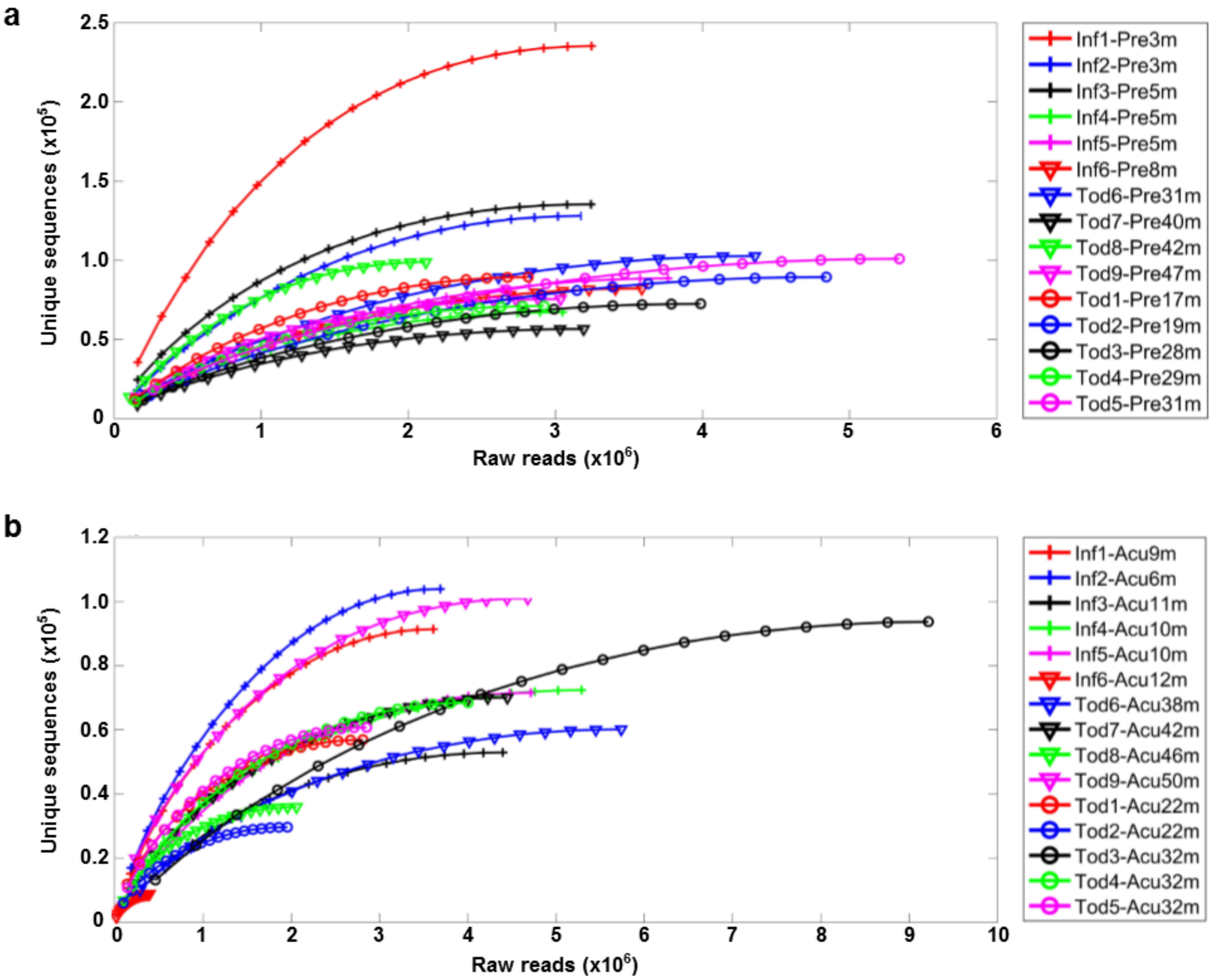

Supplementary Figure 4 Rarefaction analysis of paired PBMC malaria cohort sequencing libraries. (**a**) Pre-malaria PBMC rarefaction curves (N=15). (**b**) Acute malaria PBMC rarefaction curves (N=15). Raw reads were subsampled to varying depths, and MIDCIRS was used to determine the number of unique RNA molecules. All single-read sequences that occurred before subsampling were discarded. Single-read sequences that occurred as a results of subsampling were included as unique RNA molecules. The number of unique RNA molecules discovered saturated for all samples, indicating adequate sequencing depth.

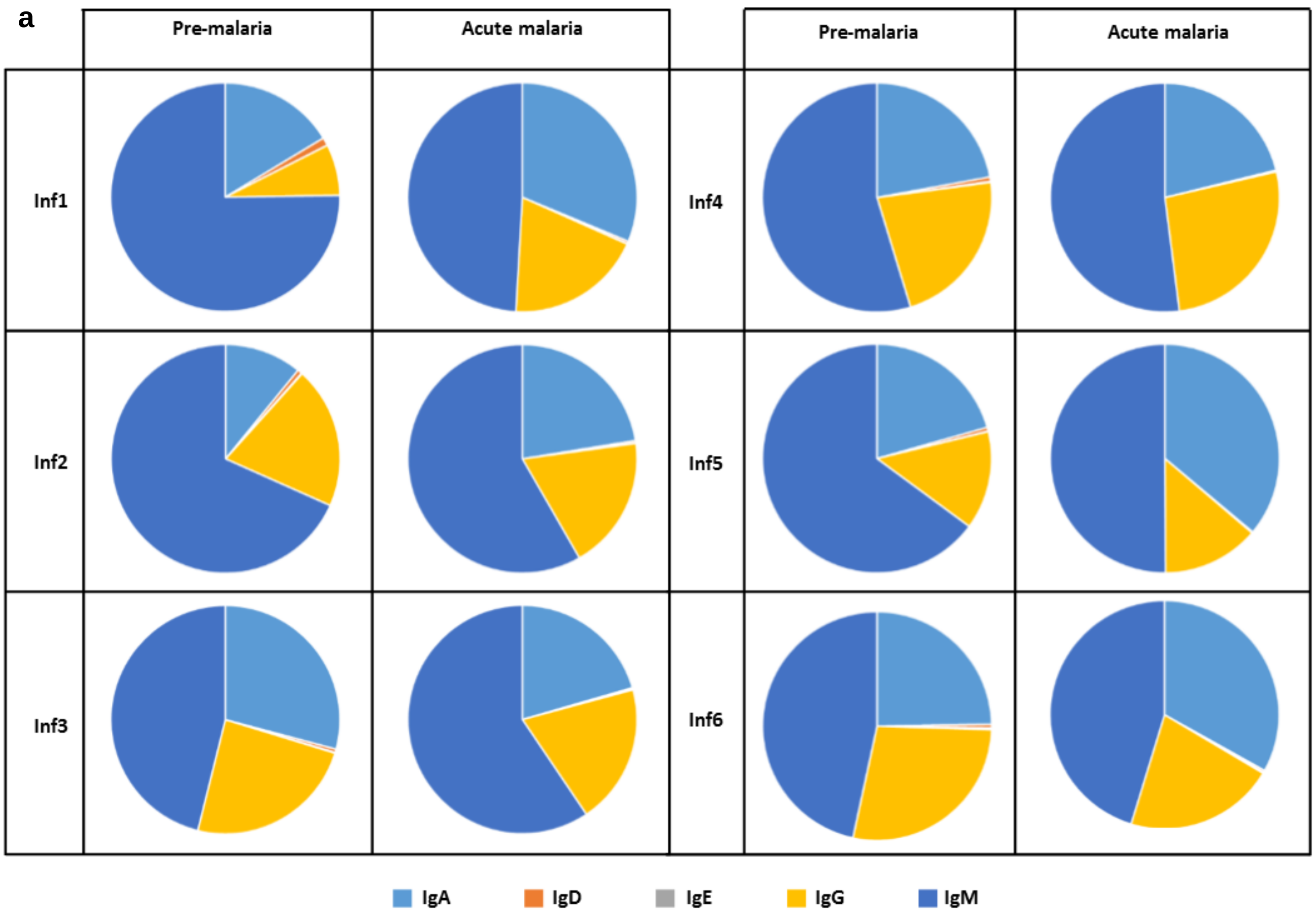
a
Pre-malaria
Acute malaria
Pre-malaria
Acute malaria
Inf1
Inf2
Inf3
Inf4
Inf5
Inf6
IgA
IgD
IgE
IgG
IgM

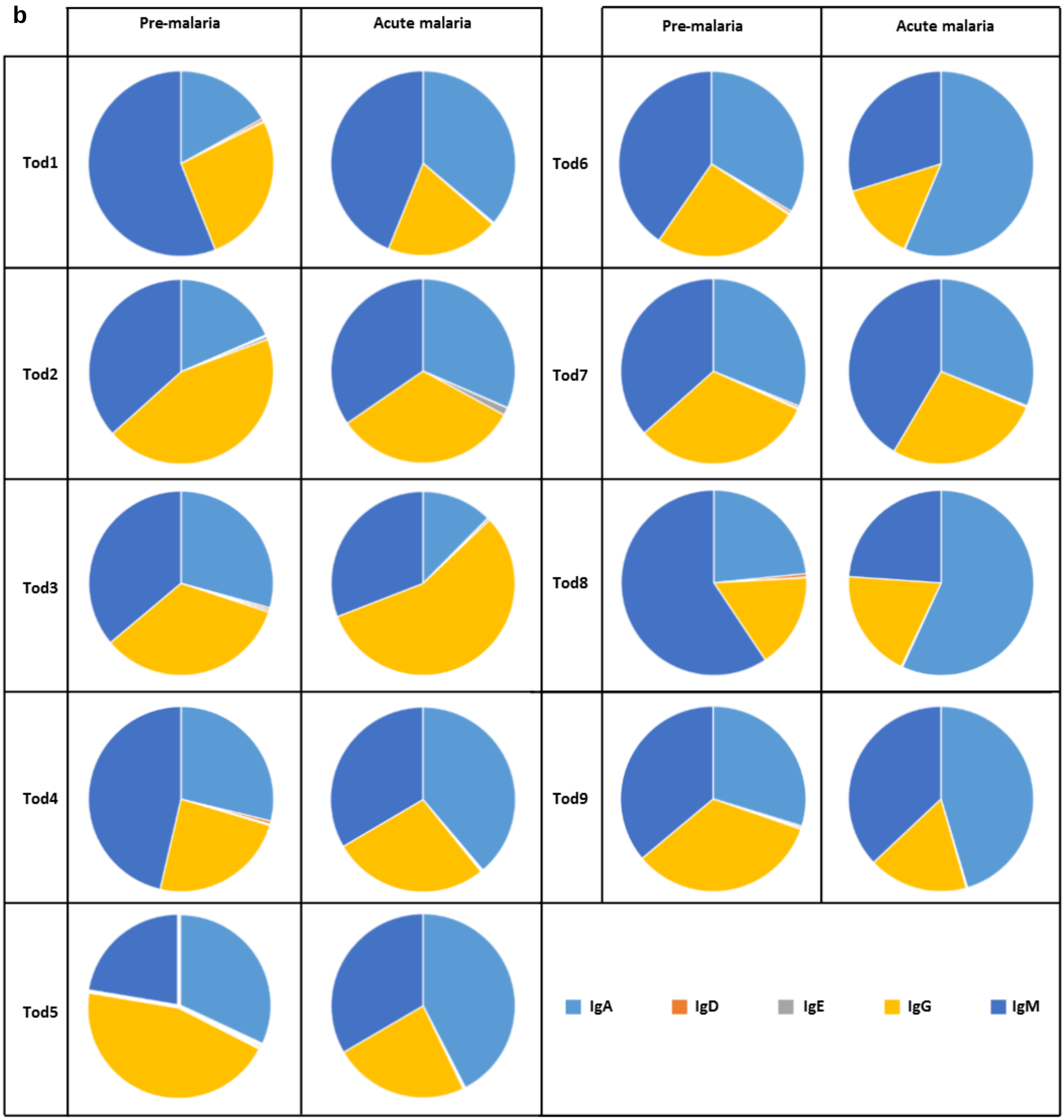

Supplementary Figure 5 Antibody isotype distribution for infants and toddlers. Antibody isotypes were assigned based on the portion of the constant region sequenced for infants (**a**) and toddlers (**b**). Isotype distribution was weighted on the number of RNA molecules.

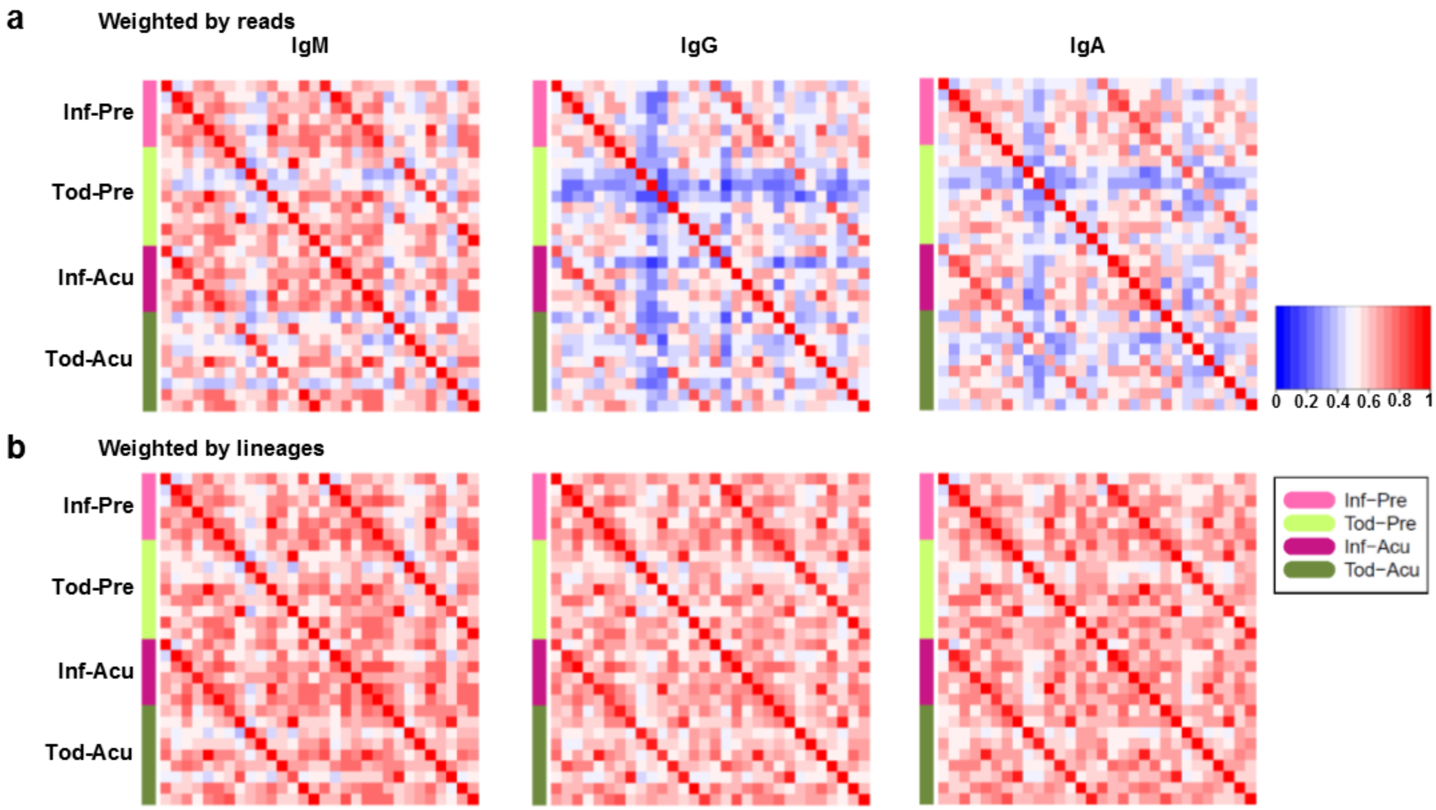

Supplementary Figure 6 Correlation between VDJ usage in paired PBMCs samples (N=15 pairs of pre-malaria and acute malaria). Correlations weighted by reads (**a**) or by lineage (**b**). The color bar left of each panel as well as in figure legend indicates the sample group: infant pre-malaria (pink), toddler pre-malaria (light green), infant acute malaria (maroon), and toddler acute malaria (dark green). Color indicates strength of Pearson correlation. The diagonal lines in each panel indicate same sample self-correlation; two shorter off-diagonal lines indicate correlations from two timepoints of the same individual.

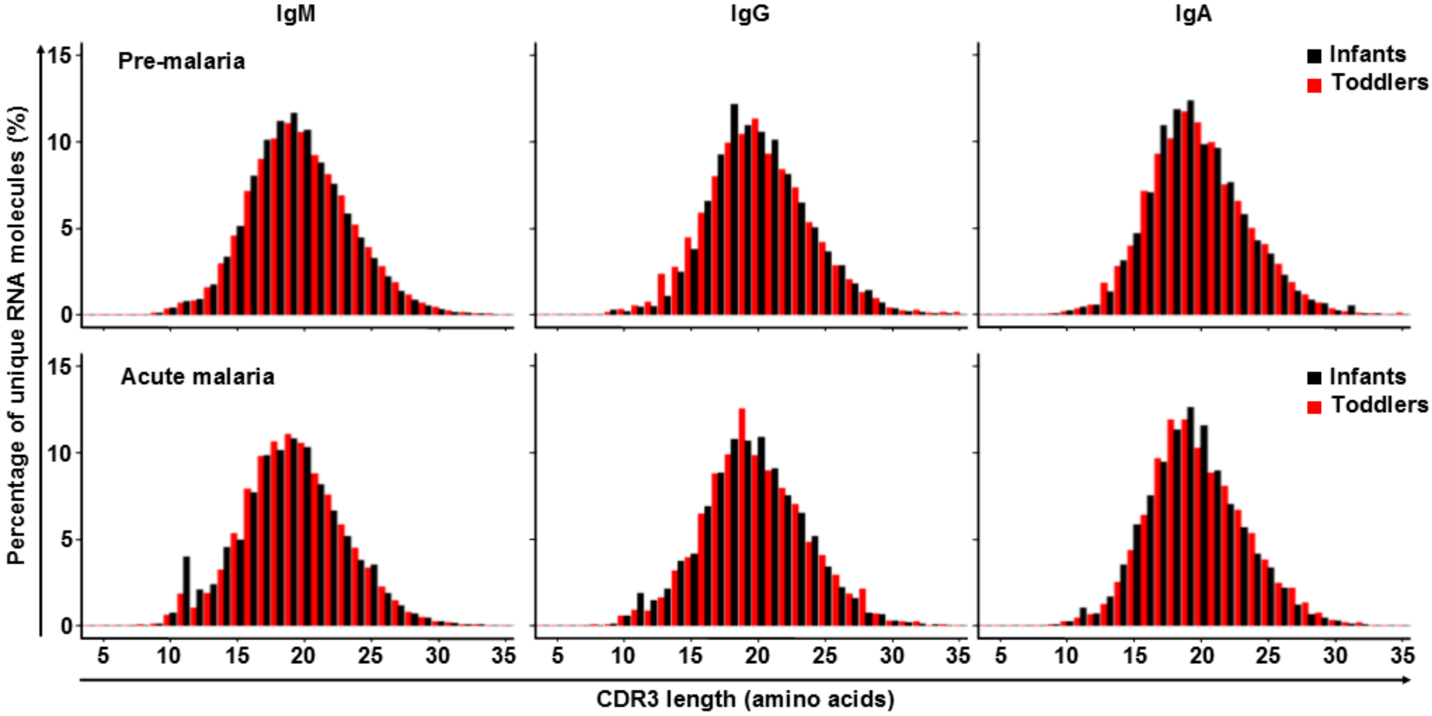

Supplementary Figure 7 CDR3 amino acid lengths of infants (black, N=6) and toddlers (red, N=9) at pre-malaria (top) and acute malaria (bottom) timepoints, separated by isotype.

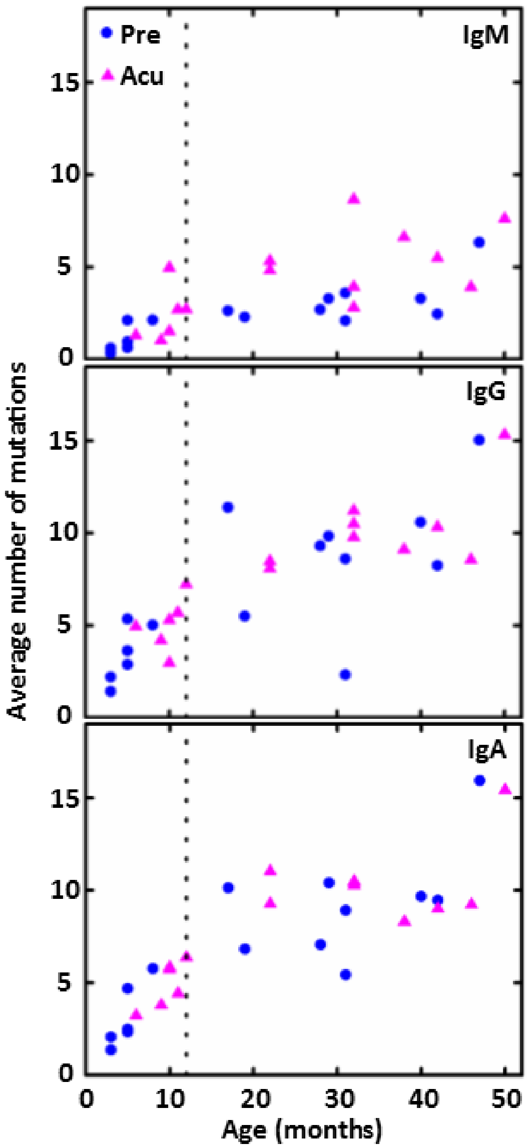

Supplementary Figure 8 Correlation between average number of mutations and age for initial, paired pre- and acute malaria samples. Initial samples (N=15) suggested a step-wise increase in SHM load around 12 months which prompted us to divide our cohort into two age groups and delve further into the antibody repertoire properties. We have since added 9 pre-malaria samples around the transition, 11 months to 17 months, which were shown in **Fig. 2b**.

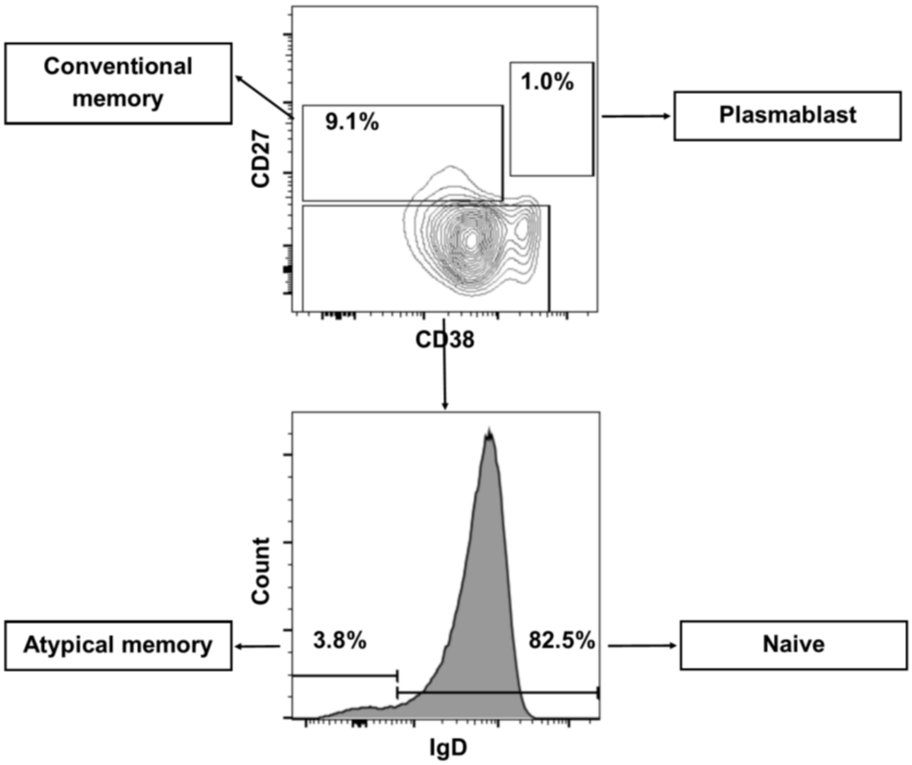

Supplementary Figure 9 Flow cytometry B cell gating and atypical memory percentage. B cells were first gated by scatter, then live, dump (CD4, CD8, CD14, CD56) negative, and then $CD19^{+}$. Conventional memory B cells ($CD20^{+}CD27^{+}$), plasmablasts ($CD27^{bright}CD38^{bright}$), and naïve B cells ($CD20^{+}CD27^{-}CD38^{low}$) were gated for further analysis. Atypical memory B cells ($CD20^{+}CD27^{-}CD38^{low}IgD^{-}$) make up a minor portion of the naïve-like B cells. Percentage of total B cells is displayed for each subpopulation.

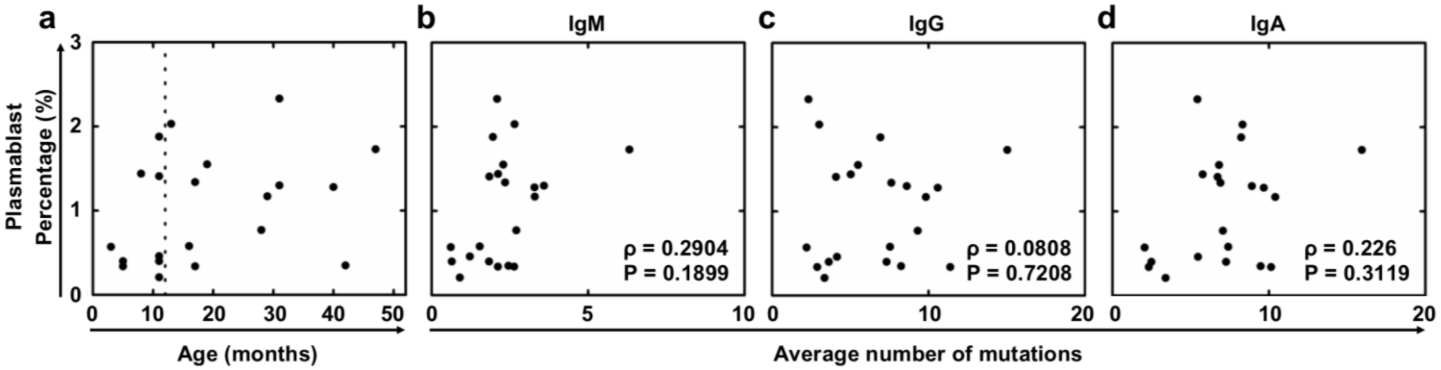

Supplementary Figure 10 Comparison between pre-malaria plasmablast percentage of total B cells and average number of mutations. (**a**) Plasmablast percentages of total B cells compared with age. (**b-d**) Plasmablast percentages of total B cells compared with average number of mutations of IgM (**b**), IgG (**c**), and IgA (**d**) sequences from bulk PBMCs in pre-malaria samples from infants (N=9) and toddlers (N=13). ρ and P values determined by Spearman's rank correlation have been listed in the figure.

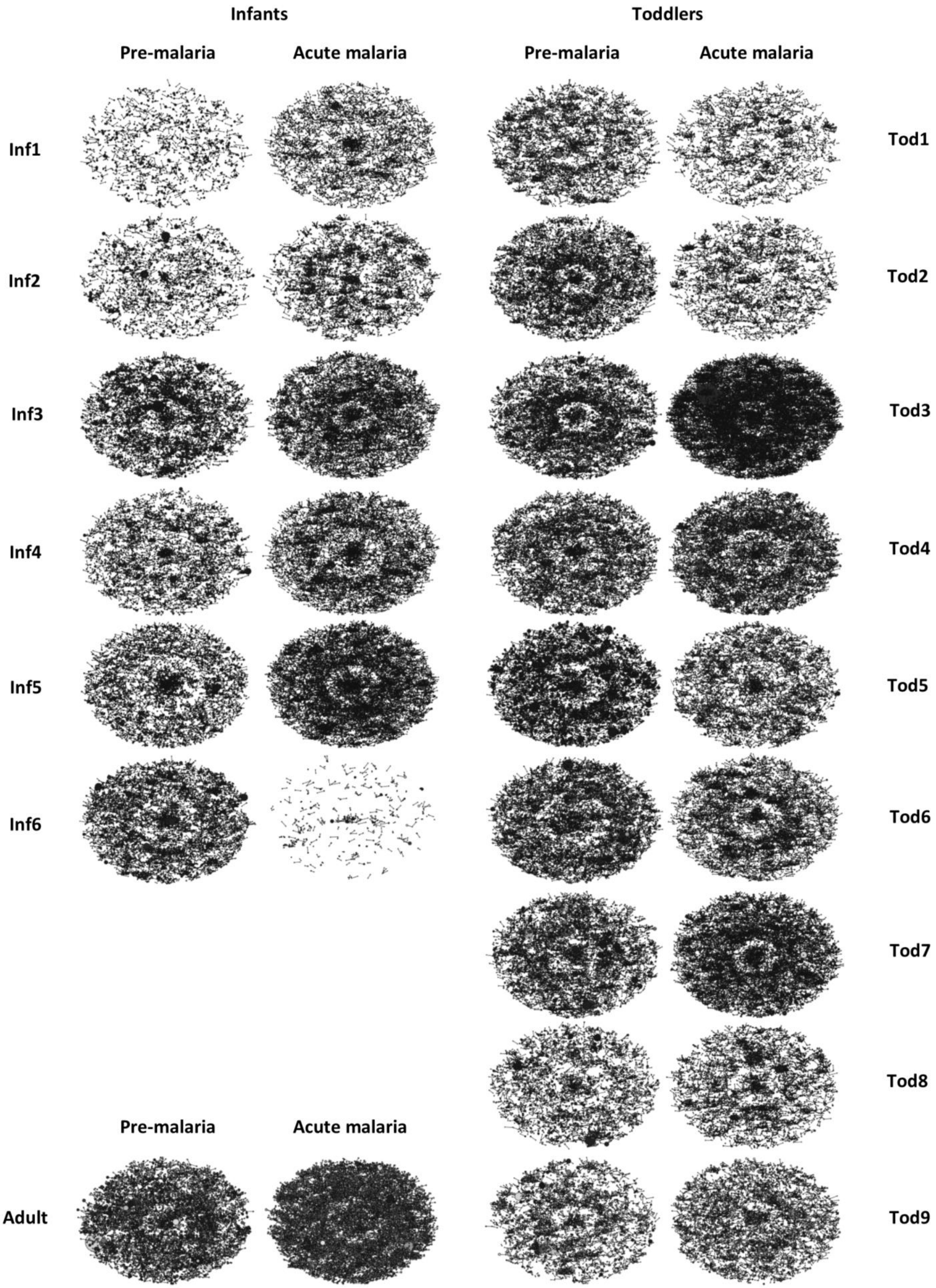
Infants
Toddlers
Pre-malaria
Acute malaria
Pre-malaria
Acute malaria
Inf1
Inf2
Inf3
Inf4
Inf5
Inf6
Tod1
Tod2
Tod3
Tod4
Tod5
Tod6
Tod7
Tod8
Tod9
Pre-malaria
Acute malaria
Adult

Supplementary Figure 11 Lineage structure visualization. Lineage distribution structures for pre-malaria and acute malaria samples for all individuals with corresponding pre-malaria and acute malaria PBMC samples. A 24 year old adult malaria patient was also included. Lineages composed of only a single unique RNA molecule were excluded. Clonal lineages shown in **Fig 5b,c** are densely packed here. Therefore, it is not intended to show intra-lineage structure for all individual lineages in each panel; rather, each panel provides an overview of all lineages for one individual at one timepoint. The darker the cluster in each oval-shaped global lineage map, the more densely packed lineages there are.

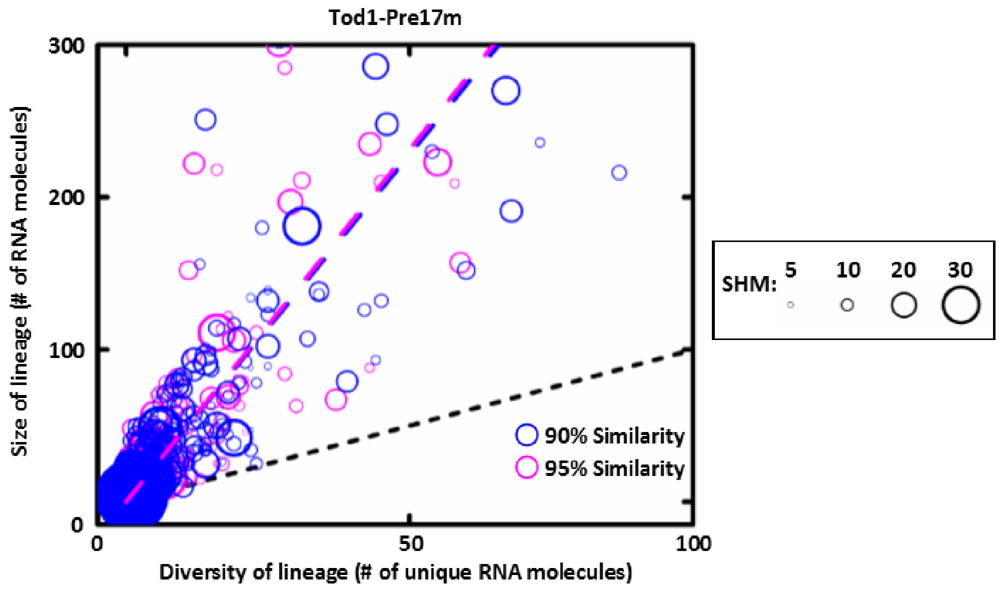

Supplementary Figure 12 Comparison between different thresholds for lineage formation. 90% (blue) and 95% (pink) nucleotide similarities of the CDR3 region were used as the threshold to generate lineages. The distribution of the size vs diversity of lineages and the linear regressions (blue and pink dashed lines) of the lineage distributions generated by the two thresholds were compared. The area of the circle corresponds to the average SHM within the lineage. Black dotted line depicts y=x parity.

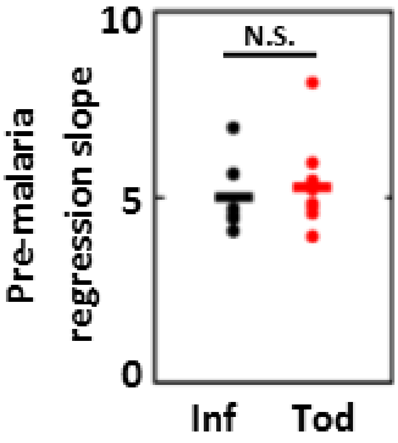

Supplementary Figure 13 Pre-malaria lineage diversification between infants and toddlers. Pre-malaria lineage size/diversity linear regression slopes (**Fig. 6a**, blue dashed lines) were compared between infants (black) and toddlers (red). N.S. indicates not significant by Mann Whitney U test, two-tailed. Bars indicate means.

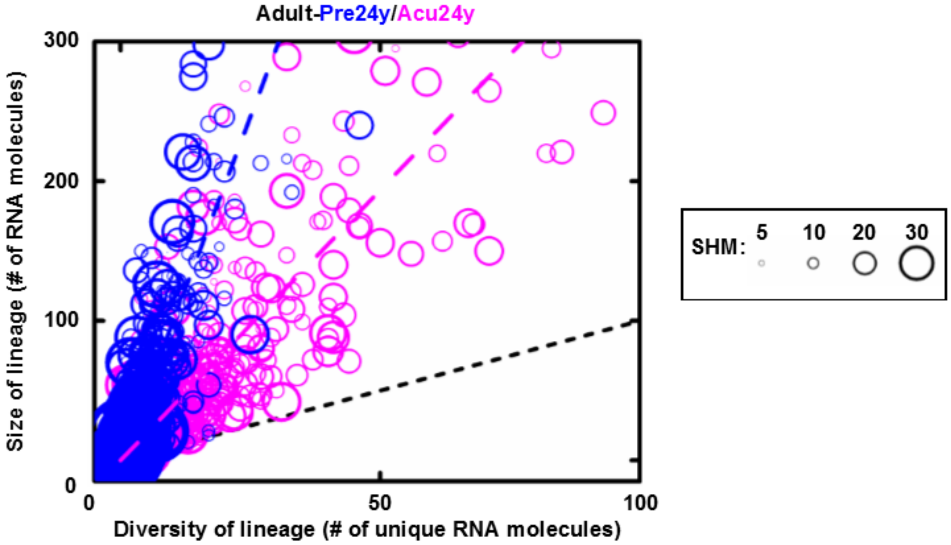

Supplementary Figure 14 Adult B cell lineage diversification. Size and diversity of B cell lineages between pre-malaria (blue) and acute malaria (pink) samples for a 24 year old adult malaria patient. Area of the circles corresponds to the average number of mutations within that lineage. Dashed lines represent the linear fit for pre- (blue) and acute (pink) lineages; black dotted line depicts y=x parity. Both axes were trimmed to be consistent with the main figures.

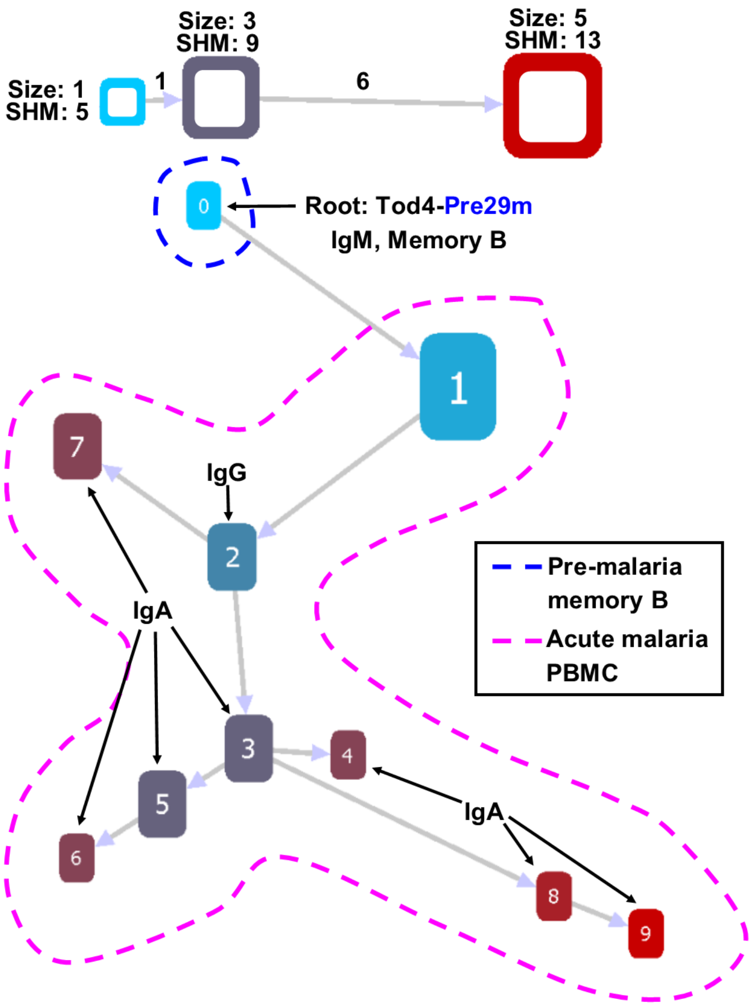

Supplementary Figure 15 Multi-timepoint shared lineage example. Intra-lineage structure for a representative lineage from **Fig. 6**. Blue dashed curve encompasses the pre-malaria timepoint derived sequence, and pink dashed curve encompasses the acute malaria timepoint derived sequences. Each node is a unique RNA molecule species. The height of the node corresponds to the number of RNA molecules of the same species, the color corresponds to the SHM load, and the distance between nodes is proportional to the Levenshtein distance between the node sequences, as indicated in the legend above the lineage. Unlabeled node shares the isotype with the root.

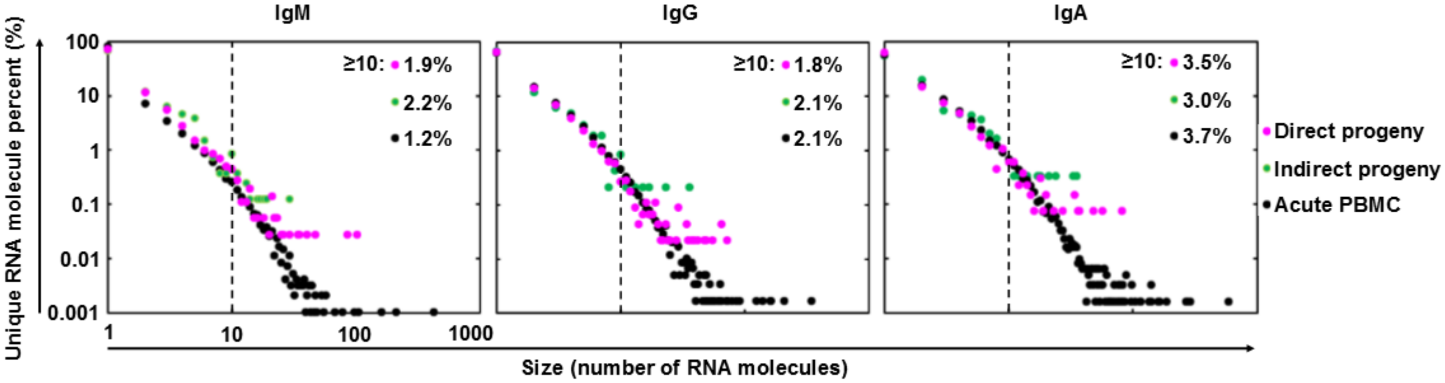

Supplementary Figure 16 Pre-malaria memory B cells' acute progeny RNA abundance. Shared lineages containing sequences from pre-malaria memory B cells and acute malaria PBMCs were formed as in **Fig. 6c-f** and **Supplementary Fig. 16**. Acute sequences from these lineages were classified as direct progeny (pink, corresponding to pink box in **Fig.6c**) if they can be traced directly back to a pre-malaria memory B cell sequence or indirect progeny (green, corresponding to acute sequences in the same lineages as the dark blue slice in **Fig. 6c**) if they cannot (i.e. they stem from a separate branch in the lineage tree). The RNA abundance distribution for these sequences were split by isotype and compared to the bulk acute PBMCs (black) from the same individuals (N=8 toddlers, Tod5 was not included because there were insufficient cells for FACS sorting). Vertical dashed line indicates 10 RNA molecule cutoff, with the percentage of unique RNA molecules larger than this cutoff displayed in the top right corner of each panel.

a

#	Sequence	Alignment
	Germline	agtggtggttactactggagctggatccgccagcacccagggaagggcctggagtggattgggtacatctattacagtgg
0	Inf-Acu11m, IgG	....
1	Inf-Acu11m, IgA	....
2	Inf-Acu11m, IgA	....
3	Inf-Acu11m, IgG	....
4	Inf-Acu11m, IgG	...................a..
5	Inf-Acu11m, IgG	...................a..
6	Inf-Acu11m, IgG	....
7	Inf-Acu11m, IgG	......a..a................
8	Inf-Acu11m, IgA	....c...
9	Inf-Acu11m, IgA	....
10	Inf-Acu11m, IgG	....a......................c...
11	Inf-Acu11m, IgA	..................g...
	Germline	gagcacctactacaacccgtccctcaagagtcgagttaccatatcagtagacacgtctaagaaccagttctccctgaagc
0	Inf-Acu11m, IgG	....
1	Inf-Acu11m, IgA	....
2	Inf-Acu11m, IgA	....a.
3	Inf-Acu11m, IgG	....g....................
4	Inf-Acu11m, IgG	....
5	Inf-Acu11m, IgG	....
6	Inf-Acu11m, IgG	....g....................
7	Inf-Acu11m, IgG	....
8	Inf-Acu11m, IgA	...................g..
9	Inf-Acu11m, IgA	...............g..................c...t...
10	Inf-Acu11m, IgG	..............g...
11	Inf-Acu11m, IgA	...................g..
	Germline	tgagctctgtgactgccgcggacacggccgtgtattac..
0	Inf-Acu11m, IgG	....
1	Inf-Acu11m, IgA	....
2	Inf-Acu11m, IgA	....
3	Inf-Acu11m, IgG	....
4	Inf-Acu11m, IgG	....
5	Inf-Acu11m, IgG	....
6	Inf-Acu11m, IgG	...................a..
7	Inf-Acu11m, IgG	....
8	Inf-Acu11m, IgA	....
9	Inf-Acu11m, IgA	....
10	Inf-Acu11m, IgG	....
11	Inf-Acu11m, IgA	............g...........t.................................t..............t......
	Germline	
0	Inf-Acu11m, IgG	ggccagggaaccctggtcaccgtctcctca
1	Inf-Acu11m, IgA	....
2	Inf-Acu11m, IgA	....
3	Inf-Acu11m, IgG	....
4	Inf-Acu11m, IgG	....
5	Inf-Acu11m, IgG	....
6	Inf-Acu11m, IgG	....
7	Inf-Acu11m, IgG	....
8	Inf-Acu11m, IgA	....
9	Inf-Acu11m, IgA	....
10	Inf-Acu11m, IgG	....
11	Inf-Acu11m, IgA	....

b

	Germline	t g g g t g c g a c a g g c c c c t g g a c a a g g g c t t g a g t g g a t g g g a t g g a t c a g c g c t t a c a a t g g t a a c a c a a a c t a t g c a c a
0	Inf3-Acu11m, IgA	. . . . g . . . . a . . . . g . . . g . . . . . . a . . . .
1	Inf3-Acu11m, IgG	. . . . g . . . . g . a . . . . g . . . g . . . . . . a . . . .
2	Inf3-Acu11m, IgG	. . . . g . . . . a . . . . g . . . c . . . . . . a . . . .
3	Inf3-Acu11m, IgA	. . . . g . . . . a . . . . g . . . g . . . . . . a . . . .
4	Inf3-Acu11m, IgG	. . . . g . . . . a . . . . g . . . g . a c . . . a . . . .
5	Inf3-Acu11m, IgA	. . . . g . . . . a . . . . g . . . g . a . . . . a . . . . c . . . .
6	Inf3-Acu11m, IgG	. . . . g . . . . a . . . . g . . t . . a c . . . a . . . .
7	Inf3-Acu11m, IgA	. . . . g . . . . a . a . . a . . . g . . . . t . . . g . . . .
	Germline	g a a g c t c c a g g g c a g a g t c a c c a t g a c c a c a g a c a c a t c c a c g a g c a c a g c c t a c a t g g a g c t g a g g a g c c t g a g a t c t g
0	Inf3-Acu11m, IgA	. . . a . . . . g . . . . t . . . .
1	Inf3-Acu11m, IgG	. . . a . . . . g . . . . t . . . .
2	Inf3-Acu11m, IgG	. . . a . . . . c . . . . g . . . . t . . . .
3	Inf3-Acu11m, IgA	. . . a . . . . g . . . . . t . t . . . . . . t . . a . . . . a . .
4	Inf3-Acu11m, IgG	. . . a . . . . g . . . . g . . . . t . . . . . . t . . . .
5	Inf3-Acu11m, IgA	. . . a . . . . g . . . . t . . . . . a a . . . . . . a . . . .
6	Inf3-Acu11m, IgG	. . . a . . . . g . . . . g . . . . t . . . . . . t . . . .
7	Inf3-Acu11m, IgA	. . . a . . . . a . . . . c . . . . c . . . . .
	Germline	a c g a c a c g g c c g t g t a t t a c . . . .
0	Inf3-Acu11m, IgA	. . . .
1	Inf3-Acu11m, IgG	. . . . t . . . .
2	Inf3-Acu11m, IgG	. . . . c . . . .
3	Inf3-Acu11m, IgA	. . . .
4	Inf3-Acu11m, IgG	. . . .
5	Inf3-Acu11m, IgA	. . . . a . . . .
6	Inf3-Acu11m, IgG	. . . . t . . . .
7	Inf3-Acu11m, IgA	. . . .
	Germline	g g c c a g g g a a c c c t g g t c a c c g t c t c c t c a
0	Inf3-Acu11m, IgA	. . . .
1	Inf3-Acu11m, IgG	. . . .
2	Inf3-Acu11m, IgG	. . . . g . . . .
3	Inf3-Acu11m, IgA	. . . .
4	Inf3-Acu11m, IgG	. . . .
5	Inf3-Acu11m, IgA	. . . .
6	Inf3-Acu11m, IgG	. . . .
7	Inf3-Acu11m, IgA	. . . .

c

	Germline	cactgggtccgccaggcttccgggaaagggctggagtgggttggccgtattagaagcaaagctaacagttacgcgacagc
0	Tod4-Pre29m, IgM	....a............
1	Tod4-Acu32m, IgM	....c................a............
2	Tod4-Acu32m, IgG	....c................a............
3	Tod4-Acu32m, IgA	....c................a............
4	Tod4-Acu32m, IgA	....c................a............
5	Tod4-Acu32m, IgA	....c................a............
6	Tod4-Acu32m, IgA	....c........c................a............
7	Tod4-Acu32m, IgA	....c.........a......a............
8	Tod4-Acu32m, IgA	....c................a............
9	Tod4-Acu32m, IgA	....c................a............
	Germline	atatgctgcgtcggtgaaaggcaggttcaccatctccagagatgattcaaagaacacggcgtatctgcaaatgaacagcc
0	Tod4-Pre29m, IgM	..tc.............c...g......
1	Tod4-Acu32m, IgM	..tc............g..t....................
2	Tod4-Acu32m, IgG	..tc............c..t....................
3	Tod4-Acu32m, IgA	..tc............c..t..t.................
4	Tod4-Acu32m, IgA	..tc............c..t..t.................
5	Tod4-Acu32m, IgA	..tc............c..t..t.................
6	Tod4-Acu32m, IgA	..tc............c..t..t.................
7	Tod4-Acu32m, IgA	..tc............c..t....................
8	Tod4-Acu32m, IgA	..tc...........ac............t.............................t....................
9	Tod4-Acu32m, IgA	..tc...........ac............t.............................t.c..................
	Germline	tgaaaaccgaggacacggccgtgtattac...
0	Tod4-Pre29m, IgM	....
1	Tod4-Acu32m, IgM	....
2	Tod4-Acu32m, IgG	....................a...........g......................g........................
3	Tod4-Acu32m, IgA	....................a...........g...
4	Tod4-Acu32m, IgA	....................a.......t...g...
5	Tod4-Acu32m, IgA	....................a...........g......................g........................
6	Tod4-Acu32m, IgA	....................a...........g......................g........................
7	Tod4-Acu32m, IgA	....................a...........g......................g........................
8	Tod4-Acu32m, IgA	..........a.........a.c.........g.......................g.......................
9	Tod4-Acu32m, IgA	..........a.........a.c.........g.......................g.......................
	Germline	ggccagggaaccctggtcaccgtctcctca
0	Tod4-Pre29m, IgM	....
1	Tod4-Acu32m, IgM	....
2	Tod4-Acu32m, IgG	....
3	Tod4-Acu32m, IgA	....
4	Tod4-Acu32m, IgA	....
5	Tod4-Acu32m, IgA	....
6	Tod4-Acu32m, IgA	....
7	Tod4-Acu32m, IgA	..............a...............
8	Tod4-Acu32m, IgA	....
9	Tod4-Acu32m, IgA	....

Supplementary Figure 17 Sequence alignment for illustrated lineages. The CDR3 region has been highlighted in yellow. The top row displays the IMGT germline allele sequence, and dashes indicate where the sequences are identical to the germline. (**a**) Corresponds to the lineage in **Fig. 5b**, (**b**) corresponds to the lineage in **Fig. 5c**, and (**c**) corresponds to the lineage in **Supplementary Fig. 15**.

Supplementary Tables

Supplementary Table 1 Primers used for antibody sequence library generation.

Primer name	Sequence (5'-3')
**RT primers**	
IgG	ACACTCTTTCCCTACACGACGCTCTTCCGATCTNNNNNNNNNNNNA AGACCGATGGGCCCTTG
IgA	ACACTCTTTCCCTACACGACGCTCTTCCGATCTNNNNNNNNNNNNG AAGACCTTGGGGCTGGT
IgM	ACACTCTTTCCCTACACGACGCTCTTCCGATCTNNNNNNNNNNNNG GGAATTCTCACAGGAGACG
IgE	ACACTCTTTCCCTACACGACGCTCTTCCGATCTNNNNNNNNNNNNG AAGACGGATGGGCTCTGT
IgD	ACACTCTTTCCCTACACGACGCTCTTCCGATCTNNNNNNNNNNNNG GGTGTCTGCACCCTGATA
**1st PCR forward primers**	
ILLUPE2LR1	GACGTGTGCTCTTCCGATCTCGCAGACCCTCTCACTCAC
ILLUPE2LR2	GACGTGTGCTCTTCCGATCTTGGAGCTGAGGTGAAGAAGC
ILLUPE2LR3	GACGTGTGCTCTTCCGATCTTGCAATCTGGGTCTGAGTTG
ILLUPE2LR4	GACGTGTGCTCTTCCGATCTGGCTCAGGACTGGTGAAGC
ILLUPE2LR5	GACGTGTGCTCTTCCGATCTTGGAGCAGAGGTGAAAAAGC
ILLUPE2LR6	GACGTGTGCTCTTCCGATCTGGTGCAGCTGTTGGAGTCT
ILLUPE2LR7	GACGTGTGCTCTTCCGATCTACTGTTGAAGCCTTCGGAGA
ILLUPE2LR8	GACGTGTGCTCTTCCGATCTAAACCCACACAGACCCTCAC
ILLUPE2LR9	GACGTGTGCTCTTCCGATCTAGTCTGGGGCTGAGGTGAAG
ILLUPE2LR10	GACGTGTGCTCTTCCGATCTGGCCCAGGACTGGTGAAG
ILLUPE2LR11	GACGTGTGCTCTTCCGATCTGGTGCAGCTGGTGGAGTC
1st PCR reverse primer	
ILLUPE1adaptor_short	ACACTCTTTCCCTACACGAC
2nd PCR reverse primer	
ILLUPE1adaptor	AATGATACGGCGACCACCGAGATCTACACTCTTTCCCTACACGAC
**2nd PCR forward primers with 7 library barcodes**	
ILLUPE2TSBC21	CAAGCAGAAGACGGCATACGAGATAACGAAACGTGACTGGAGTTC AGACGTGTGCTCTTCCGATCT
ILLUPE2TSBC22	CAAGCAGAAGACGGCATACGAGATAACGTACGGTGACTGGAGTTC AGACGTGTGCTCTTCCGATCT
ILLUPE2TSBC23	CAAGCAGAAGACGGCATACGAGATAACCACTCGTGACTGGAGTTC AGACGTGTGCTCTTCCGATCT
ILLUPE2TSBC25	CAAGCAGAAGACGGCATACGAGATAAATCAGTGTGACTGGAGTTC AGACGTGTGCTCTTCCGATCT
ILLUPE2TSBC26	CAAGCAGAAGACGGCATACGAGATAAGCTCATGTGACTGGAGTTC AGACGTGTGCTCTTCCGATCT
ILLUPE2TSBC27	CAAGCAGAAGACGGCATACGAGATAAAGGAATGTGACTGGAGTTC AGACGTGTGCTCTTCCGATCT
ILLUPE2TSBC28	CAAGCAGAAGACGGCATACGAGATAACTTTTGGTGACTGGAGTTC AGACGTGTGCTCTTCCGATCT

Supplementary Table 2 Sequencing read statistics for control libraries.

Library	Number of cells	Number of raw reads	Number of merged reads	Number of Ig reads	Number of reads truncated to 320bp	Number of useful MIDs[a]	Percentage of Reads in useful MIDs	Number of useful MIDs containing more than one sub-group[b]
Libraries for naive B cells from healthy controls	1,000	46,320	22,742	9,201	9,149	797	94.30	1
	2,000	44,846	18,602	17,421	17,267	2,176	93.29	2
	10,000	228,711	99,370	62,242	61,121	7,102	94.73	9
	20,000	293,279	196,570	184,754	182,818	23,991	93.27	49
	100,000	1,153,763	1,074,771	1,048,523	1,041,048	165,663	92.63	1,137
	200,000	2,191,738	2,107,762	2,059,944	2,045,047	404,225	91.41	7,239
	1,000,000	7,494,809	7,342,163	7,258,253	7,207,962	1,516,098	86.44	108,172

[a] A useful MID has more than two reads. If there are only two reads in a MID, they are discarded unless they are identical.
[b] The number of MIDs containing more than one type of antibody heavy chain transcripts**.**

Supplementary Table 3 Cohort and cell type availability.

Patient	Pre-malaria				Acute malaria		
	Pre-Index	Pre- Age	PBMC	Memory B	Acute-Index	Acute Age	PBMC
Inf1	Inf1-Pre3m	3m	Yes	I.S.	Inf1-Acu9m	9m	Yes
Inf2	Inf2-Pre3m	3m	Yes	J.F.	Inf2-Acu6m	6m	Yes
Inf3	Inf3-Pre5m	5m	Yes	I.S.	Inf3-Acu11m	11m	Yes
Inf4	Inf4-Pre5m	5m	Yes	J.F.	Inf4-Acu10m	10m	Yes
Inf5*	Inf5-Pre5m	5m	Yes	J.F.	Inf5-Acu10m	10m	Yes
Inf6	Inf6-Pre8m	8m	Yes	J.F.	Inf6-Acu12m	12m	Yes
Inf7	Inf7-Pre11m	11m	Yes	Yes	N.A.	N.A.	N.A.
Inf8	Inf8-Pre11m	11m	Yes	Yes	N.A.	N.A.	N.A.
Inf9	Inf9-Pre11m	11m	Yes	Yes	N.A.	N.A.	N.A.
Inf10	Inf10-Pre11m	11m	Yes	Yes	N.A.	N.A.	N.A.
Inf11	Inf11-Pre11m	11m	Yes	Yes	N.A.	N.A.	N.A.
Tod1*	Tod1-Pre17m	17m	Yes	Yes	Tod1-Acu22m	22m	Yes
Tod2	Tod2-Pre19m	19m	Yes	Yes	Tod2-Acu22m	22m	Yes
Tod3†	Tod3-Pre28m	28m	Yes	Yes	Tod3-Acu32m	32m	Yes
Tod4	Tod4-Pre29m	29m	Yes	Yes	Tod4-Acu32m	32m	Yes
Tod5	Tod5-Pre31m	31m	Yes	J.F.	Tod5-Acu32m	32m	Yes
Tod6	Tod6-Pre31m	31m	Yes	Yes	Tod6-Acu38m	38m	Yes
Tod7†	Tod7-Pre40m	40m	Yes	Yes	Tod7-Acu42m	42m	Yes
Tod8	Tod8-Pre42m	42m	Yes	Yes	Tod8-Acu46m	46m	Yes
Tod9	Tod9-Pre47m	47m	Yes	Yes	Tod9-Acu50m	50m	Yes
Tod10	Tod10-Pre13m	13m	Yes	Yes	N.A.	N.A.	N.A.
Tod11	Tod11-Pre16m	16m	Yes	Yes	N.A.	N.A.	N.A.
Tod12	Tod12-Pre17m	17m	Yes	Yes	N.A.	N.A.	N.A.
Tod13	Tod13-Pre17m	17m	Yes	Yes	N.A.	N.A.	N.A.

I.S. indicates insufficient PBMC for FACS sorting or analysis.
J.F. indicates just flow cytometry analysis.
N.A indicates samples were not available.
* Same individual
† Same individual

Supplementary Table 4 Sequencing read statistics of paired PBMCs from the malaria cohort.

Sample	PBMCs[a]	Raw reads	Mapped reads	Percent Mapped	Unique RNA molecules
Inf1-Pre3m	3,000,000	3,246,180	2,989,252	92.1%	41,842
Inf1-Acu9m	3,000,000	3,608,436	3,348,589	92.8%	32,800
Inf2-Pre3m	3,000,000	3,176,623	2,987,587	94.0%	35,379
Inf2-Acu6m	3,000,000	3,689,115	3,481,675	94.4%	29,523
Inf3-Pre5m	4,150,000	3,242,619	3,070,458	94.7%	37,234
Inf3-Acu11m	5,000,000	4,396,739	4,153,830	94.5%	42,634
Inf4-Pre5m	5,000,000	3,048,762	2,810,018	92.2%	45,445
Inf4-Acu10m	3,700,000	5,287,767	4,864,629	92.0%	29,694
Inf5-Pre5m*	5,000,000	3,764,663	3,425,015	91.0%	54,516
Inf5-Acu10m*	50,00,000	4,712,120	4,374,600	92.8%	41,774
Inf6-Pre8m	5,000,000	3,588,177	3,456,165	96.3%	47,254
Inf6-Acu12m	400,000	395,765	378,182	95.6%	03,447
Tod1-Pre17m*	5,000,000	2,816,309	2,576,372	91.5%	53,551
Tod1-Acu22m*	1,380,000	2,811,617	2,593,849	92.3%	12,514
Tod2-Pre19m	5,000,000	4,842,338	4,673,875	96.5%	40,600
Tod2-Acu22m	1,920,000	1,956,906	1,886,521	96.4%	15,285
Tod3-Pre28m†	5,000,000	3,988,677	3,687,883	92.5%	35,567
Tod3-Acu32m†	5,000,000	9,218,255	8,565,149	92.9%	47,144
Tod4-Pre29m	5,000,000	2,924,629	2,851,964	97.5%	48,950
Tod4-Acu32m	5,000,000	4,004,416	3,846,197	96.0%	40,628
Tod5-Pre31m	5,000,000	5,338,867	5,126,888	96.0%	31,531
Tod5-Acu32m	3,000,000	2,853,984	2,736,902	95.9%	26,955
Tod6-Pre31m	5,000,000	4,356,975	4,198,929	96.4%	44,665
Tod6-Acu38m	2,170,000	5,738,001	5,460,964	95.2%	22,270
Tod7-Pre40m†	5,000,000	3,192,503	2,893,482	90.6%	34,901
Tod7-Acu42m†	4,740,000	4,448,008	4,079,432	91.7%	34,185
Tod8-Pre42m	5,000,000	2,120,127	2,058,164	97.1%	48,939
Tod8-Acu46m	2,100,000	2,060,234	1,986,239	96.4%	17,039
Tod9-Pre47m	3,000,000	3,035,618	2,682,991	88.4%	20,094
Tod9-Acu50m	3,000,000	4,678,879	3,912,981	83.6%	18,447

[a]Number of PBMCs differs because of the age dependent blood draw volume and cell recovery.
* Same individual
† Same individual

Supplementary Table 5 Percentage of unique RNA sequences assigned to novel alleles for each sample. Novel alleles detected by TIgGER and our method were combined.

Sample	Percentage of Unique RNA sequences assigned to novel germline alleles
Inf1-Pre3m	4.81%
Inf1-Acu9m	6.21%
Inf2-Pre3m	8.44%
Inf2-Acu6m	9.11%
Inf3-Pre5m	1.78%
Inf3-Acu11m	4.91%
Inf4-Pre5m	11.83%
Inf4-Acu10m	9.63%
Inf5-Pre5m*	8.19%
Inf5-Acu10m*	7.72%
Inf6-Pre8m	6.02%
Inf6-Acu12m	6.79%
Tod1-Pre17m*	9.82%
Tod1-Acu22m*	7.51%
Tod2-Pre19m	2.54%
Tod2-Acu22m	2.34%
Tod3-Pre28m†	16.91%
Tod3-Acu32m†	15.05%
Tod4-Pre29m	3.61%
Tod4-Acu32m	4.80%
Tod5-Pre31m	6.98%
Tod5-Acu32m	6.79%
Tod6-Pre31m	5.89%
Tod6-Acu38m	4.15%
Tod7-Pre40m†	18.30%
Tod7-Acu42m†	13.84%
Tod8-Pre42m	7.40%
Tod8-Acu46m	5.71%
Tod9-Pre47m	13.10%
Tod9-Acu50m	13.15%

* Same individual
† Same individual

Supplementary Table 6 Average mutation number of naïve B cells.

Sample	Number of naive B cells	Average number of mutations
Inf1-Acu9m	10000	0.31
Inf2-Pre3m	10000	0.20
Inf3-Pre5m	10000	0.29
Inf4-Pre5m	10000	0.27
Inf5-Pre5m*	10000	0.40
Tod1-Pre17m*	10000	0.79
Tod2-Pre19m	10000	0.57
Tod3-Pre28m†	10000	0.53
Tod4-Pre29m	100000	1.07
Tod7-Pre40m†	10000	0.45
Tod8-Pre42m	100000	1.20

* Same individual
† Same individual

Supplementary Table 7 Replacement and silent mutations and their ratio for PBMCs in infants and toddlers.

			FWR			CDR			Average R/S Ratio	
			R	S	R/S Ratio	R	S	R/S Ratio	FWR	CDR
Infant	Pre	IgM	0.54	0.11	4.98	0.18	0.04	5.15	3.00 ± 1.12	5.54 ± 0.25
		IgG	1.54	0.70	2.21	1.36	0.24	5.67		
		IgA	1.48	0.65	2.28	1.29	0.22	5.75		
	Acute	IgM	1.36	0.34	4.05	0.58	0.11	5.52		
		IgG	1.88	0.85	2.22	1.62	0.30	5.35		
		IgA	2.03	0.90	2.25	1.75	0.30	5.79		
Toddler	Pre	IgM	1.12	0.35	3.20	0.58	0.11	5.54	2.41 ± 0.45	5.34 ± 0.25
		IgG	3.42	1.57	2.17	2.73	0.54	5.05		
		IgA	3.88	1.82	2.14	3.15	0.58	5.41		
	Acute	IgM	2.16	0.79	2.73	1.33	0.24	5.44		
		IgG	4.28	2.02	2.11	3.39	0.68	5.02		
		IgA	4.33	2.04	2.12	3.55	0.64	5.59		

Nucleotide mutations resulting in amino acid substitutions (Replacement, R) or no amino acid substitutions (silent, S) in the framework region (FWR2 and 3) and complementary determining regions (CDR1 and 2) of infants (N=6) and toddlers (N=9), weighted by unique RNA molecules. CDR3 and FWR4 were not included in this analysis due to the difficulty determining the germline sequence. FWR1 for all sequences was also omitted because it was not covered entirely by some of the primers. Average displayed as mean ± standard deviation.

Supplementary Table 8 Pre-malaria and acute malaria shared lineage count.

Patient	Shared lineages	Unique memory B cell Sequences	Containing pre-malaria memory B cells
Inf1	29	N.A.	N.A.
Inf2	131	N.A.	N.A.
Inf3	215	N.A.	N.A.
Inf4	142	N.A.	N.A.
Inf5	214	N.A.	N.A.
Inf6	83	N.A.	N.A.
Tod1	308	3,423	149
Tod2	385	7,856	145
Tod3†	1230	6,023	926
Tod4	1194	5,073	209
Tod5	260	N.A.	N.A.
Tod6	346	6,363	111
Tod7†	472	4,771	161
Tod8	581	2,399	98
Tod9	414	2,534	135

The number of lineages containing sequences from both the pre-malaria and acute malaria timepoints. For malaria-experienced individuals with 10,000 FACS sorted pre-malaria memory B cells available, the number of unique memory B cell sequences and two-timepoint-shared lineages that contain sequences from the sorted memory B cells from the pre-malaria timepoint.
N.A. indicates not applicable
† Same individual
